# Prostate cancer: advancements in targeted therapies

**DOI:** 10.3389/fendo.2026.1924299

**Published:** 2026-10-09

**Authors:** Ilesha Ewart, Manreet Randhawa

**Affiliations:** Beatson West of Scotland Cancer Centre, Glasgow, United Kingdom

**Keywords:** antibody drug conjugate (ADC), bispecific T-cell engager (BiTE), mCRPC, metastatic hormone-sensitive prostate cancer, MHSPC, prostate cancer, radioligand therapy (RLT), targeted therapies prostate cancer

## Abstract

Prostate cancer is the most common cancer diagnosed in men. Metastatic castration-resistant prostate cancer (mCRPC) is associated with significant morbidity and mortality. The last decades have seen the introduction of multiple new therapeutic agents, such as androgen receptor pathway inhibitors, PARP inhibitors, new cytotoxic agents, and radioligand therapies. Further advancements are being made with immunotherapy utilising new molecular targets. As these treatments move earlier in the treatment pathway, determining the optimal treatment sequencing becomes the challenge. This review aimed to highlight established treatments, recent developments, and promising therapies in mCRPC.

## Introduction

Prostate cancer is the most common cancer diagnosed in men and the second most common cause of cancer death for men in the UK (1). The incidence of prostate cancer is rising primarily due to an ageing population, but also due to an increase in awareness (2). In men with metastatic prostate cancer, the lethal form of the disease, castration resistance inevitably develops.

Metastatic castration-resistant prostate cancer (mCRPC) has significant morbidity and mortality, with a median survival of 12–36 months following diagnosis (3, 4). Until the 2010s, taxane chemotherapy in the form of docetaxel was the only licensed treatment. Further novel hormone therapies targeting the androgen receptor (AR) signalling pathways known as androgen receptor pathway inhibitors (ARPIs) were later introduced. However, after pivotal trials, taxanes and ARPIs are now often given earlier in the disease course and in selected patients, used in combination for men with metastatic hormone-sensitive prostate cancer (mHSPC) (5–8).

For this reason, it is essential to identify further treatment options for mCRPC. The development of targeted and immune-mediated therapies has increased the treatment armamentarium for prostate cancer, allowing a more personalised approach in some patients.

## Androgen receptor pathway inhibitors

The AR is a key driver of prostate cancer progression and therefore lends itself to a target in drug development. Despite the use of androgen deprivation therapy (ADT) in the form of gonadotrophin-releasing hormone (GnRH) analogues, progression to mCRPC is inevitable, which generally occurs within 2–3 years (9). This is thought to be partially due to aberrant AR signalling driven by androgen precursors derived from the adrenal glands, alongside AR overexpression, AR splicing variants, and the accumulation of mutations (10, 11).

The introduction of ARPIs represented a significant advancement in the treatment of prostate cancer. The AR comprises three domains: the N-terminal domain, the DNA-binding domain, and the ligand-binding domain (LBD). Enzalutamide, darolutamide, and apalutamide competitively inhibit androgens from binding to the LBD, inhibiting nuclear translocation, whereas abiraterone is a potent inhibitor of CYP17, which is required for androgen biosynthesis.

In AFFIRM, enzalutamide was shown to significantly increase the overall survival (OS) in patients with mCRPC who had progressed following docetaxel (18.4 *vs*. 13.6 months) (12). In PREVAIL, enzalutamide was compared with placebo in chemotherapy-naive mCRPC patients (13). Final analysis showed a median OS of 36 months in the enzalutamide group *vs*. 31 months in the placebo group (14). This showed that, despite 68% of the patients in the placebo arm receiving subsequent ARPI, first-line enzalutamide still provided a significant benefit.

CYP17 is found in prostatic, testicular, and adrenal tissue and is responsible for the catalysis of androgen synthesis from cholesterol and glucocorticoids (15). Abiraterone blocks CYP17 and, thus, androgen synthesis. The COU-AA-301 trial randomised patients with mCRPC previously treated with docetaxel to abiraterone and prednisolone or placebo. At a median follow-up of 20.2 months, the OS was 15.8 months in the abiraterone group *vs*. 11.2 months in placebo, and abiraterone improved all secondary outcomes (16). COU-AA-302 compared abiraterone and placebo in mCRPC patients without previous chemotherapy. The radiographic progression-free survival (rPFS) was 16.6 *vs*. 8.3 months (17), and final analysis demonstrated a significantly longer OS (34.7 *vs*. 30.3 months) (18).

Both enzalutamide and abiraterone can also be used in the castrate-sensitive setting. LATITUDE showed that abiraterone is efficacious in high-risk mHSPC (6). STAMPEDE demonstrated that abiraterone plus ADT had significantly longer OS than ADT alone in men with metastatic, node-positive, or high-risk locally advanced disease (19). PEACE-1 showed that the combination of ADT, abiraterone, and docetaxel in newly diagnosed mHSPC improved the OS and radiographic progression free survival (rPFS) with a manageable safety profile (20). Similarly, ENZAMET showed that the use of enzalutamide with ADT in mHSPC significantly improved both PSA and clinical PFS with a hazard ratio (HR) of 0.67 *versus* ADT plus first-generation nonsteroidal antiandrogens (5).

As ARPIs become more commonly used in the hormone-sensitive setting, the question of optimum treatment sequencing remains. While it is observed in clinical practice, there is limited evidence to support the use of second-line ARPIs in patients previously treated with another. Khalaf et al. (21) showed in a crossover trial that abiraterone and enzalutamide had similar first-line outcomes, but that only 4% of patients had a PSA response (defined as >30% decrease from baseline) to second-line abiraterone *vs*. 36% to second-line enzalutamide. Despite this, real-world evidence suggests that sequential ARPI–ARPI treatment is still used in countries where access is not an issue (22).

### ARPI resistance

Despite the efficacy of ARPIs, nearly all patients develop secondary resistance. Over 50% of patients with mCRPC have AR gene amplifications (23), which are associated with ARPI resistance and poor prognosis (24). There is evidence that AR alterations develop in patients who initially respond to ARPIs, suggesting that this could contribute to acquired treatment failure and to primary resistance (25). Several studies have shown that patients with AR amplifications have worse outcomes on ARPI treatments (26, 27). However, some studies have also shown that AR amplification is associated with longer time to clinical progression (28). A potential confounding factor of these studies is the use of liquid biopsy. AR copy number gain is correlated with higher cell-free DNA levels. As these are associated with higher tumour burden, it introduces challenges in interpreting the prognostic value of AR amplifications.

AR mutations are infrequent in treatment-naive patients, more common in mCRPC, and are present in up to 40% of patients previously treated with an ARPI (23). They are associated with poorer outcomes and are commonly found in sites of metastatic disease and after ARPI treatment, suggesting that they may be preferentially expressed in previously hormone-treated patients (28, 29). Truncated AR splice variants lack the LBD where ARPIs bind, but retain the DNA-binding domain and the nuclear localisation signal. This can result in transcription and downstream signalling without AR binding, which allows tumour cells to proliferate even in the presence of ADT.

The most common variant is AR splice variant 7 (AR-V7). Preclinical models have shown that enzalutamide and abiraterone resistance is associated with the presence of AR-V7 (30). This is suspected to be due to the lack of an LBD, the direct target of enzalutamide and the indirect target of abiraterone. The presence of AR-V7 could therefore be used to guide treatment decisions, as its presence indicates that patients may respond better to taxane-based therapy rather than to an ARPI. Graf et al. showed that patients with AR-V7 had superior survival with taxanes compared with second-line APRIs, while AR-V7-negative patients had superior survival on second-line ARPIs *versus* taxanes (31). However, this cross-sectional study was open to selection bias as treating physicians were more likely to choose taxane therapy for patients with more advanced or aggressive disease. There is additional evidence that AR-V7 acts a transcriptional repressor of tumour suppressor genes (32).

Targeted agents against AR-V7 are now being tested in early-phase clinical trials. Niclosamide is an anti-helminthic medication that has been shown to be active against a variety of tumour types. Its mechanism in prostate cancer is thought to be due to degrading AR variants or its implication in other pathways, such as phosphoinositide 3-kinase/protein kinase B/mammalian target of rapamycin (PI3K/AKT/mTOR) (33). Niclosamide has poor oral bioavailability, with the initial phase I trial assessing its combination with enzalutamide being abandoned after dose-limiting toxicities at a non-therapeutic plasma level (34). However, a recent phase Ib trial showed that a reformulated version could be tolerated at therapeutic levels and safely combined with abiraterone and prednisolone. Of the eight patients evaluated, four had a PSA50 (at least a 50% reduction in PSA) response and two a durable response to treatment (35). A phase II trial looking at this combination is currently in progress (36).

Mutated AR may have increased binding affinity and allow the AR signalling pathway to be activated by lower-potency androgens, other steroid hormones, and AR antagonists (37). This is another driver of prostate cancer progression and treatment resistance. A potential treatment approach is therefore to disrupt androgen production at the source. CYP11a1 is a catalyst of the multistep pathway by which cholesterol is converted to testosterone, and its inhibition fundamentally stops the synthesis of all steroid hormones. In the phase I/II CYPIDES study, opevesostat, a CYP11a1 inhibitor, demonstrated anti-tumour activity, with 24 out of 45 patients in phase II achieving PSA50 (38). The most significant toxicity was adrenal suppression, a function of steroid blockade, which was manageable once established on appropriate steroid replacement. Ongoing studies include OMAHA-3, which evaluates opevesostat *vs*. ARPI switch in patients previously treated with an ARPI and chemotherapy (39), and OMAHA-4, which assesses opevesostat *vs*. ARPI switch in chemotherapy-naive mCRPC patients stratified for LBD mutation (40).

### PI3K/AKT/mTOR inhibitors

PI3K is a family of enzymes that regulate anabolic and catabolic cellular activities. Aberrations in the PI3K/AKT/mTOR pathway are present in up to 40% and 70% of prostate cancers in the early and late stages, respectively (41). Studies support AR–PI3K pathway interactions, with the theory that the suppression of AR signalling via ADT could select for tumour cells with PI3K activation and repressed AR activity, resulting in progression to castration-resistant prostate cancer (CRPC) (42). Initial research therefore focused on patients with CRPC who had progressed on first-line ARPIs, assessing whether PI3K inhibitors could help overcome resistance.

Despite the theoretical mechanisms described, there has been minimal benefit observed with the addition of PI3K pathway inhibitors. Hotte et al. (43) showed that the addition of a PI3K inhibitor to abiraterone in patients with CRPC had no objective or PSA response. Similarly, Armstrong et al. (44) showed that PI3K inhibitor plus enzalutamide had no effect on PFS. It should be noted that both trials continued first-line ARPI past progression. However, de Bono et al. (45) showed that ipatasertib, an AKT inhibitor, in combination with abiraterone improved the rPFS and OS in patients previously treated with docetaxel. A few patients in this study had prior ARPI exposure. Given this, further studies in the hormone-sensitive prostate cancer (HSPC) setting and with pre-selected patients were designed.

Phosphatase and tensin homolog deleted on chromosome 10 (PTEN) is a tumour suppressor that negatively regulates the PI3K pathway. PTEN deficiency is associated with advanced prostate cancer and poor outcomes (46). Cuzick et al., in localised disease, found that PTEN loss was significantly associated with mortality and remained a strong predictor of poor outcomes even in patients classified as low-risk; however, no prognostic value was found in high-risk patients (47). In mCRPC, PTEN loss has been shown to be associated with poorer outcomes in patients on abiraterone after taxane-based therapy (48). PTEN loss could therefore be used to inform prognostic models and active surveillance strategies in the localised setting and could potentially identify patients with more aggressive disease in the mCRPC setting.

Preclinical models suggested that, in PTEN-deficient disease, the combination PI3K/AKT/mTOR and AR inhibition produced better outcomes than single-pathway inhibition, thought to be due to the crosstalk between the two pathways (49). CAPitello-280 evaluated capivasertib (an AKT inhibitor) plus docetaxel *vs*. docetaxel plus placebo in chemotherapy-naive mCRPC patients whose disease had progressed on ARPI. However the study was discontinued as interim analysis suggested that it was unlikely to meet its dual endpoints of rPFS and OS (50). CAPItello-281 randomised patients with PTEN-deficient HSPC to receive capivasertib or placebo plus abiraterone, prednisolone, and ADT. The results showed a significant difference in rPFS (33.2 *vs*. 25.7 months) with a manageable safety profile (51). These results suggest that, in the PTEN-deficient population, combination therapy with AKT inhibitors is a promising new treatment avenue.

### PARP inhibitors

PARP inhibitors (PARPi) act on DNA damage repair pathways. There are two main pathways of DNA damage repair, depending on the mechanism of damage. Single-strand breaks are repaired by base excision repair, nucleotide excision repair, or mismatch repair. Double-strand breaks are repaired by homologous recombination repair (HRR) or non-homologous end joining. Approximately 30% of prostate cancers are affected by DNA repair alterations (52). Some of the genes affected include *BRCA1*, *BRCA2*, *ATM*, *CHEK2*, *PALB2*, *MSH2*, and *MSH6*, with *BRCA2* and *ATM* mutations being the most commonly observed (53, 54). Tumours with these gene alterations are unable to be repaired by the HRR pathway. Synthetic lethality describes cell death due to the combination of a mutated gene, for example *BRCA*, with another inactivated gene or its product. By inhibiting PARP in patients with a *BRCA* mutation, there is increased degradation of single-strand to double-strand breaks, leading to eventual cell death. This is thought to be due to multiple mechanisms, including obstruction of replication forks *(*55*).*

TOPARP-B is a randomised phase II trial examining the efficacy of olaparib in mCRPC patients with a known pathogenic mutation in an HRR gene. Its intent was to validate the predictive gene panel for PARPi, with the results suggesting a dose-dependent relationship (56). The PROfound trial looked at mCRPC patients with genetic alterations in *BRCA1*, *BRCA2*, or *ATM* who experienced disease progression on enzalutamide or abiraterone (57). These patients were randomised to receive olaparib in the treatment arm or abiraterone or enzalutamide (whichever they had not received prior) in the standard of care (SOC) arm. In patients with a genetic mutation, rPFS was significantly longer in the olaparib group (7.4 *vs*. 3.6 months), with an overall response rate (ORR) of 33%.

*In vivo* models have shown potential synergistic effects between ARPIs and PARPi. Suggested mechanisms include the induction of a functional HRR deficiency by ARPI inhibition of the downstream transcriptional activity of the AR and the inhibition of the AR signalling pathway by inhibiting PARP recruitment of the AR to its transcription site (58). Three pivotal phase III trials looked at ARPI/PARPi combinations in mCRPC.

TALAPRO-2 assessed the combination of talazoparib and enzalutamide as first-line treatment for mCRPC. Patients were permitted to have had docetaxel or abiraterone in the mHSPC setting. Patients were divided into an HRR-unselected cohort and an HRR-mutated cohort. Final analysis showed that, in the HRR-mutated cohort, OS significantly improved in the treatment arm (45.8 *vs*. 37.0 months), and rPFS was 33.1 *vs*. 19.5 months (59).

PROpel randomised unselected mCRPC patients to olaparib or placebo in combination with abiraterone and prednisolone. Patients were treatment-naive in the setting of mCRPC, but could have had docetaxel or alternative ARPI in the hormone-sensitive setting. Testing for HRR mutations was done after randomisation. The initial analysis favoured the olaparib group, with rPFS of 24.8 months *vs*. 16.6 months. The OS at the final analysis favoured the experimental arm (42.1 *vs*. 34.7 months), but was not statistically significant (60).

MAGNITUDE evaluated niraparib or placebo combined with abiraterone and prednisolone, with patients allowed up to a 4-month lead-in of abiraterone. Patients were tested for HRR mutations and randomised 1:1 to the experimental and control arms. The final analysis showed a clinically significant benefit in time to symptomatic progression and time to cytotoxic chemotherapy, but no significant OS benefit (30.36 *vs*. 28.55 months) (61).

There is now also evidence for its use earlier in the disease course. AMPLITUDE evaluated niraparib and abiraterone plus prednisolone *versus* placebo in mHSPC with HRR gene alterations. This showed a significant improvement in rPFS, particularly in the *BRCA* subgroup (median not yet reached *vs*. 26 months). The OS data are not yet mature (62). More recently, TALAPRO-3 compared talazoparib and enzalutamide *vs*. placebo plus enzalutamide as first-line treatment in patients with mHSPC. The initial analysis showed an rPFS benefit in all patients regardless of *BRCA* mutations. In the *BRCA*-mutated subgroup, the 3-year rPFS was 77.2% in the treatment arm *vs*. 48.8% in placebo, with an HR of 0.368. In the *BRCA* non-mutated subgroup, the 3-year rPFS was 76.2% *vs*. 60.2%, with an HR of 0.567 (63).

## Radioligand therapies

Radioligand therapies (RLTs) have been used in other tumour types, but are fairly novel in the field of prostate cancer. It is a targeted treatment that combines a therapeutic radionucleotide with a tumour-specific ligand. Prostate-specific membrane antigen (PSMA) is a transmembrane glycoprotein that is highly expressed in prostate cancer cells, with low levels of expression in normal tissues. Over 95% of primary prostate cancers express moderate to high levels of PSMA, and increasing PSMA expression levels have been shown to correlate with disease progression in mCRPC (64). While the utility of PSMA in prostate cancer remains the most significant, it has also been described in the neovasculature of multiple solid organ tumours such as breast, ovarian, kidney, and sarcoma (65–67).

The overexpression of PSMA in prostate cancer acts as a target in nuclear medicine imaging. PSMA positron emission tomography (PET) scans utilise a radiotracer, commonly 68Ga-PSMA-11, to assess for the presence of metastatic disease, either before definitive therapy has commenced in intermediate- or high-risk patients or in patients who have a biochemical recurrence.

The discovery of PSMA allowed for the development of precision-targeted therapies in the field of theranostics. The extracellular domain allows for ligand binding and ligand-induced internalisation. This led to the development of treatments that selectively bind PSMA-positive cells to deliver localised treatment, which can be in the form of radiation, cytotoxics, or immunotherapeutic agents.

177Lutetium (Lu)-PSMA-617 (^177^Lu-PSMA-617) is a RLT that delivers targeted beta particles to tumour cells expressing PSMA, utilising the ligand vipivotide tetraxetan. The VISION and PSMAfore trials showed promising results using ^177^Lu-PSMA-617 in mCRPC, with the former showing a significant improvement in both the primary endpoint of imaging-based PFS and OS (with HRs of 0.4 and 0.62, respectively) (68). Similarly, PSMAfore reported a HR of 0.41 for imaging-based PFS; however, this did not translate into an OS benefit (69).

While these results are promising, concerns have been raised regarding the limitations of the trial designs. VISION included patients with mCRPC who had progressed after at least one ARPI and one line of taxane chemotherapy; importantly, patients could be enrolled only if they were deemed unsuitable for second-line cabazitaxel. There were 153 patients in the control arm, and post-protocol, 53 patients received second-line cabazitaxel, raising the question of whether these patients were truly unfit for second-line taxane. In addition, over half of the patients in the SOC arm had already received two ARPIs and one taxane, meaning that they would be randomised to a treatment they had received previously. PSMAfore included patients with mCRPC progressing after one ARPI and no previous chemotherapy. The SOC arm allowed only ARPI, ADT, and supportive care. This does not reflect the current SOC, which would suggest that treatment with docetaxel should be considered rather than an ARPI re-challenge.

In the hormone-sensitive setting, PSMAddition, a phase III trial, was conducted randomising patients into ^177^Lu-PSMA-617 in addition to SOC (ARPI and ADT) or SOC alone. The initial analysis showed a significant improvement in rPFS in the treatment arm, with a HR of 0.72. The OS data have not yet matured (70).

Further challenges include equity of access to PSMA PET and RLTs. The results of cost-effectiveness studies (71, 72) vary and are based in high-income Western countries; thus, the results are not applicable to low- and middle-income countries. Data from early-access compassionate-use programmes support use in a real-world population, but with the data, again, largely from the United States, Australia, and Western Europe (73). In practice, this technology remains out of reach for many due to lack of access and cost barriers.

The other RLT with growing interest is alpha-emitting therapies such as actinium-225. As alpha particles have higher energy, these are able to induce double-stranded DNA damage (74) and also have a shorter range, therefore limiting the radiation effects to surrounding healthy tissues (75). An established treatment option in mCRPC with bone-only involvement is radium-223 dichloride (Ra-223). This is not regarded as a true RLT, but is an alpha-emitting radiopharmaceutical. Radium is a calcium mimetic that binds to osteoblastic or sclerotic bone metastases, allowing the high-energy alpha particles to induce cell death. The ALSYMPCA trial compared Ra-223 *vs*. SOC in mCRPC patients with symptomatic bone metastases. The results favoured the treatment arm, with a median OS of 14.9 *vs*. 11.3 months and an HR of 0.7 (76). Following approval of Ra-223, ERA-223 assessed the combination of Ra-223 and abiraterone acetate plus prednisolone. However, it was discontinued due to the high rate of fractures in the treatment arm (77). A similar trial in PEACE-3 evaluated Ra-223 plus enzalutamide *vs*. enzalutamide monotherapy in patients with progressive mCRPC. The final analysis showed a significant OS benefit, with a median of 38.2 *vs*. 32.6 months, respectively, and an HR of 0.76 (78).

Although there are no completed phase 3 alpha-emitting therapy trials, the meta-analysis of retrospective studies by Ninatti et al. (79) showed promising results, with encouraging PSA50 compared with standard treatments, such as docetaxel and enzalutamide, in the same setting. However, it is important to remember that these data are largely collected from retrospective studies and, as such, cannot be compared with randomised controlled trial (RCT) data. Prospective trials evaluating alpha-emitting RLTs are ongoing (80, 81).

### Immunotherapy

Immunotherapy has been shown to have limited efficacy in prostate cancer. This is thought to be due to a suppressive tumour microenvironment with few tumour-infiltrating lymphocytes, an abundance of immunosuppressive cells such as regulatory T cells and myeloid-derived suppressor cells, and a low neoantigen burden (82). Overcoming this requires a deeper understanding of the underlying mechanisms and exploring combination treatments to make prostate cancer more susceptible to immunotherapy.

Strategies to convert the tumour microenvironment include the triggering of a pro-inflammatory response, enabling a more effective immune response. Radiotherapy and oncolytic viruses are thought to expose tumour antigens and trigger improved T-cell trafficking via localised inflammatory signals. In prostate cancer, both low-dose radiotherapy and RLTs are candidates for combination with immunotherapy. However, trials combining immune checkpoint inhibitors (ICIs) with hypofractionated radiotherapy in patients with mCRPC who had progressed on taxane therapy showed no significant difference in OS (83). Currently, PRESERVE-006 is underway, which analyses the combination of ^177^Lu vipivotide tetraxetan with an anti-CTLA4 antibody in patients with mCRPC who have progressed after ARPI. An updated analysis shows a preliminary efficacy with a manageable safety profile. Phase II data are awaited (84).

### Checkpoint inhibitors

For certain subsets of patients with specific characteristics, ICIs have been shown to be of benefit. At present, the only Food and Drug Administration (FDA)-approved ICI is pembrolizumab given in microsatellite instability high (MSI-H) prostate cancer, which represents approximately 3% of all prostate cancers (85). This is based on Keynote-158, which evaluated single-agent pembrolizumab in patients with non-colorectal MSI-H cancer. It showed promising results, with an ORR of 33.8% (86) and rPFS and OS of 4.1 and 23.5 months, respectively (87). However, only 14 patients with prostate cancer were represented in the study. A case series of prostate cancer patients reported a variable PSA50 response of between 44% and 75% (88). Keynote-199 evaluated monotherapy pembrolizumab in mCRPC patients with programmed death-ligand 1 (PD-L1)-positive, PD-L1-negative, and bone-predominant disease. While the ORR was modest at 5%, these patients had a durable response (89).

### Bispecific T-cell engagers

Studies examining the combination of ICIs with other agents have so far been disappointing (90). Research into the use of immunotherapy in mCRPC has therefore explored ways to regulate the tumour microenvironment to make prostate cancer cells more susceptible to these treatments and to utilise targets specific to the tumour. A promising avenue of research is the use of bispecific T-cell engager (BiTE) therapies (**Table 1**). These monoclonal antibodies consist of two linked single-chain variable fragments that have specificity for a tumour-associated antigen and an immune cell component, such as CD3, connected by a flexible linker.

**Table 1:** Summary of bispecific T-cell engager (BiTE) trials.

Study	Report date	Phase	Enrolment (*n*)	Antibody target	Disease setting	Primary endpoint	PSA50	rPFS	Toxicities (≥grade 3)
Hummel et al. (92)	2020	I	47	PSMA	Advanced CRPC, which has failed at least 1 taxane chemotherapy and refractive to ARPI or refused standard treatment	Safety	26%	NA	Anaemia, lymphopenia, infection
Dorff et al. (94)	2024	I	133 (dose exploration, *n* = 77; dose expansion, *n* = 56)	PSMA	Progressive mCRPC refractory to at least 1 ARPI and failed/unsuitable for taxane regime	Safety and maximum tolerated dose	30.4% (dose expansion cohort)	3.7 months (dose expansion cohort)	CRS, anaemia, hypophosphataemia, hypertensions, neutropenia, LFT derangement
Kelly et al. (96)	2024	I	97	STEAP1	Progressive mCRPC refractory to ARPI and progression following 1–2/unsuitable for taxanes	Safety and maximum tolerated dose	49%		Anaemia, myalgia, fatigue, CRS
Sumey et al. (98)	2026	I	39 (expansion cohort, *n* = 20)	STEAP1	Progressive mCRPC) refractory to ARPI and have failed at least 1 (but not more than 2) taxane regimen	Safety and tolerability as monotherapy and in combination with abiraterone acetate and enzalutamide	58% (expansion cohort)	10.5 months (expansion cohort)	Myalgia, CRS, anaemia
Stein et al. (101)	2025	I	174	KLK2	Progressive mCRPC previously treated with ≥1 line of treatment (ARPI or chemotherapy)	Safety and recommended dose	33.9%	4.4 months	Neutropenia, lymphopenia, fatigue
Patel et al. (103)	2026	Ib	51	KLK2	Progressive mCRPC—previous treatment variable dependent on cohort	Safety and determine recommended phase II regimen	64.7%	NA	Safety profile consistent with docetaxel alone

*PSMA*, prostate membrane-specific antigen; *STEAP1*, six-transmembrane epithelial antigen of the prostate 1; *KLK2*, human kallikrein 2; *mCRPC*, metastatic castration-resistant prostate cancer; *rPFS*, radiographic progression-free survival; *LFT*, liver function tests; *CRC*, cytokine release syndrome.

#### Prostate-specific membrane antigen

The first BiTE trialled in prostate cancer was pasotuxizumab, which targeted PSMA and CD3. When administered as subcutaneous injections, the development of anti-drug antibodies significantly decreased drug exposure and necessitated a switch to continuous intravenous infusion (91, 92). While this did show a PSA response, the poor pharmacokinetics and the need for continuous infusion limited its potential, and further drug development was halted.

To overcome this, subsequent BiTEs added structures to increase molecular stability and size, extending the half-life. These include bonding two BiTEs, including an Fc region or the addition of an albumin receptor. These changes have created multiple BiTEs with structural variations that now allow for more practical administration as an intermittent intravenous or subcutaneous injection.

Acapatamab is a half-life extended BiTE that also binds to PSMA and CD3. *In vitro*, it has been shown to induce potent, specific killing of PSMA-expressing prostate cancer cells (93). Dorff et al. (94) evaluated its use in a phase 1 trial with 43 patients with mCRPC refractory to ARPI and taxane chemotherapy. PSA50 responses were observed in 30.4% of patients, with a median rPFS of 3.7 months and a manageable safety profile.

#### Six-transmembrane epithelial antigen of the prostate 1

Six-transmembrane epithelial antigen of the prostate 1 (STEAP-1) is another protein currently being explored as a potential target in prostate cancer. It is a transmembrane cell surface protein that is highly overexpressed in prostate cancer, but with low or no expression in normal tissues (95). Xaluritamig is a STEAP1-targeting BiTE that showed a promising PSA50 response of 49% in a phase 1 trial (96), with the most common adverse events being cytokine release syndrome (CRS), myalgia, and fatigue. The phase 3 XALute trial, which compares xaluritamig against cabazitaxel or a second ARPI in patients who have progressed on first-line taxane chemotherapy (97), has now completed recruitment and results are awaited. Further studies are assessing its combination with ARPIs. As the phase 1 study of xaluritamig and abiraterone in chemotherapy-naive patients showed promising anti-tumour activity with a manageable safety profile (98), the phase 3 XaLience trial is currently underway (99).

#### Human kallikrein 2

Human kallikrein 2 (KLK2) is a novel therapeutic target produced and highly expressed by prostate epithelial cells, but with minimal expression in normal tissue (100). Pasritamig, a KLK2-targeting BiTE, has been shown to achieve durable responses and rPFS comparable to historic data (101). The phase III comPAS study is currently assessing pasritamig in patients who have exhausted all other available treatments (102). In addition to monotherapy, a phase Ib trial looking at combination with docetaxel has produced promising results, with a PSA50 of 64.7% and a PSA90 (estimated pooled proportion of patients with greater than a 90% reduction in the PSA levels) of 39.2% and a safety profile consistent with the established profile of docetaxel monotherapy (103).

### Antibody–drug conjugates

Antibody–drug conjugates (ADCs) are a well-established treatment in other malignancies. They combine the specificity of monoclonal antibodies with the toxicity of small-molecule payloads. The antibodies target antigens overexpressed on cancer cells, allowing for payload release in response to antibody degradation or intracellular triggers. The payload and the antibody are connected by a linker that allows for stability while in circulation and the specific release of the payload into target tissue. Research at present includes PSMA, STEAP1, TROP2, and B7-H3 as potential antigens, with over 50 trials currently ongoing in prostate cancer (**Table 2**) (104).

**Table 2:** Summary of antibody–drug conjugate (ADC) trials.

Study	Report date	Phase	Enrolment (*n*)	Antibody target	Payload	Disease setting	Primary endpoint	PSA50	rPFS	Toxicities (≥ grade 3)
Milowski et al. (106)	2016	I/II	62	PSMA	DM1	mCRPC	Sustained PSA50	8%	Not available	Peripheral neuropathy—dose-limiting
Petrylak et al. (107)	2019	I	52	PSMA	MMAE	Progressive mCRPC previously treated with docetaxel	Safety, pharmacokinetics and preliminary anti-tumour effects	14%	Not available	Neutropenia, peripheral neuropathy
Shen et al. (108)	2023	I/II	24	PSMA	Amberstatin 269	Progressive mCRPC previously treated with ≥2 FDA-approved treatments	Safety	33%	Not available	Lymphopenia, thrombocytopenia
Danila et al. (109)	2019	I	77	STEAP1	MMAE	Progressive mCRPC with no more than 2 prior lines of cytotoxic chemotherapy in metastatic setting	Safety	14%	Not available	Transaminitis, peripheral neuropathy, hyperglycaemia, hypophosphatemia
Lang et al. (116)	2022	II	20	TROP2	SN-38	Progressive mCRPC while on second-generation ARPI	PSA50 response, 6-month progression-free survival rate	0%	45%	Neutropenia
Von Amsberg et al. (117)	2025	I	9	TROP2	SN-38	TROP2-positive aggressive variant PC with exhausted standard therapy options	Preclinical and clinical activity	NA	44% (at 6 months)	Not available
De Bono et al. (125)	2024	II	181	B7-H3	Duocarmycin	Progressive mCRPC previously treated with 1 ARPI (prior docetaxel allowed)	Investigator assessed PFS	41%	51% in the low-dose arm and 59% in the high-dose arm (in patients on study >6 months or discontinued)	5 fatal TEAEs—not further specified
Doi et al. (126)	2022	I/II	127 (mCRPC, *n* = 42)	B7-H3	TOP1i	Advanced CRPC (adenocarcinoma only), unresectable or metastatic head and neck SCC, oesophageal SCC, squamous and adenocarcinoma NSCLC, SCLC, bladder cancer, sarcoma, endometrial cancer, melanoma	Safety, tolerability, and anti-tumour effect	Not available	rPFS not reported, disease response rate 73.8% in CRPC	G3 not specified—nausea, vomiting, and infusion-related reaction most common

*PSMA*, prostate membrane-specific antigen; *STEAP1*, six-transmembrane epithelia antigen of the prostate 1; *TROP2*, trophoblast antigen 2; *MMAE*, monomethyl auristatin E; *TOP1i*, topoisomerase inhibitor; *mCRPC*, metastatic castration-resistant prostate cancer; *rPFS*, radiographic progression-free survival; *LFT*, liver function tests; *TEAE*, treatment-emergent adverse event; *CRPC*, castration-resistant prostate cancer; *SCC*, squamous cell carcinoma; *NSCLC*, non-small cell lung cancer; *SCLC*, small cell lung cancer.

#### Prostate-specific membrane antigen

The first PSMA-targeted ADC was MLN2704, which carried DM1 (an anti-microtubule agent) as the cytotoxic payload. In a phase I trial, it was shown to demonstrate anti-tumour efficacy, with two of nine patients treated with the highest dose achieving PSA50 (105). However, in a phase I/II expansion, it showed limited efficacy with significant rates of peripheral neuropathy (106) and development was stopped. This was thought to be due to its narrow therapeutic window and issues with the stability of the linker.

PSMA ADC utilises monomethyl auristatin E (MMAE), an anti-microtubule agent, as its cytotoxic payload. A phase I trial showed anti-tumour activity, with 50% of the 52 patients showing a durable PSA reduction (107). The subsequent trial enrolled patients with mCRPC who had progressed after ARPI. Of the patients, 14% achieved PSA50, and 78% with measurable circulating tumour cells (CTCs) had a decline of more than 50%. However, there were also limitations due to off-target toxicity, specifically neurotoxicity and neutropenia, which led to its discontinuation.

APEX-01 evaluated ARX517, which is conjugated to amberstatin 269, a potent tubulin inhibitor. A total of 24 patients were enrolled in the dose escalation phase, with a median of four prior lines of treatment. No dose-limiting toxicities were reported at the highest dose, and an anti-tumour effect was observed (108). Enrolment for the dose expansion phase is ongoing.

#### Six-transmembrane epithelial antigen of the prostate 1

Vandortuzumab vedotin, an anti-STEAP1 agent linked to MMAE, has been assessed in an early-phase study including 77 patients with mCRPC. Of the patients, 11 achieved a PSA50, 2 showed a partial response, and 24 had stable disease (109). Drug development was halted due to its limited clinical efficacy and dose-limiting side effects.

Despite its discontinuation, vandortuzumab vedotin illustrated STEAP1 as a viable target for ADCs, prompting the development of other similar agents. Currently, ADRX-0405, ABBV-969, and DXC-008 are the anti-STEAP1 ADCs under investigation. ADRX-0405 and ABBC-969 utilise a topoisomerase inhibitor (TOP1i), a DNA damage inducer, as the payload, while DXC-008 utilises a tubulysin B analogue (110–112). ABBV-969 and DXC-008 target both STEAP1 and PSMA, with the aim of increasing the tumour coverage and therapy durability.

#### Trophoblast antigen 2

Trophoblast antigen 2 (TROP2) is a membrane-bound glycoprotein expressed in multiple epithelial cancers (113) and is associated with poorer outcomes in prostate cancer (114). It may also play a role in neuroendocrine prostate cancer, a known mechanism of ADT resistance (115).

Sacituzumab govitecan (SG), a TROP2-targeting ADC with an SN-38 payload, is an approved treatment for triple-negative breast cancer. Despite its efficacy in other cancer types, it is yet to find its position in the treatment paradigm for mCRPC. Preliminary results from a phase II trial showed no PSA50 responses in 20 patients, but with 45% reaching an rPFS of 6 months (116). It may be better explored in aggressive variant (ARV) and neuroendocrine prostate cancer due to TROP2 expression. Von Amsberg et al. showed that SG has promising clinical activity in heavily pretreated ARV patients, with 44% and 33% of patients achieving partial remission or stable disease at radiological follow-up of 3 and 6 months, respectively (117).

Datopotamab deruxtecan (Dato-DXd) carries the payload DXd, a TOP1 inhibitor. It has demonstrated clinical activity in metastatic breast cancer in early-phase trials with a lower incidence of adverse effects than SG (118, 119) and is now FDA-approved in breast and non-small cell lung cancer. Results are awaited of TROPION-PanTumour 03, a phase II trial evaluating Dato-DXd in patients with advanced solid organ tumours, including mCRPC (120).

Another example of a TROP2 ADC is ESG401, an anti-TROP2 antibody linked to a TOP1i inhibitor. Trial enrolment is ongoing, but with the preliminary results suggesting a tolerable safety profile and promising anti-tumour efficacy, with an ORR of 34.2% (121).

#### B7-H3

B7-H3 is a cell surface immunomodulatory glycoprotein that is overexpressed in prostate cancers (122). It promotes tumour invasion via the suppression of T-cell proliferation and activity (123) and is often associated with AR dysregulation (124).

MGC018 is composed of an anti-B7-H3 antibody and a duocarmycin (an ultrapotent DNA alkylating agent) payload and was assessed in the phase II TAMARACK trial. However, due to fatal adverse events, further drug development was discontinued (125).

DS-7300 carries a DXd payload. A phase I/II trial with patients with advanced malignancy unselected for B7-H3 showed evidence of a durable tumour response. Of the 127 patients enrolled, 42 had mCRPC, and 73.8% of these showed evidence of disease control (126). Updated analysis looked at 73 patients with mCRPC; these patients were heavily pretreated with a median of six previous lines of treatment. The ORR of evaluable patients was 15%, with an average PFS of 4.8 months (127). The efficacy of DS-700 in unselected, heavily pretreated mCRPC shows the potential of targeted potent cytotoxics and warrants ongoing investigation. Ongoing studies are still actively recruiting.

## Conclusion and future directions

Treatment strategies continue to evolve for mCRPC as the use of therapies is brought earlier in the disease pathway. With the development of multiple new promising drugs, more recently, PARP inhibitors, radioligand treatments, antibody–drug conjugates, and bispecific T-cell engagers, the treatment armamentarium expands and the focus shifts towards a personalised approach. The utilisation of blood, tissue, and imaging biomarkers routinely in the future is key in ensuring that optimal treatment choices are made in both settings of the disease to prolong disease control and survival.
